# Her Voice Lingers on and Her Memory Is Strategic: Effects of Gender on Directed Forgetting

**DOI:** 10.1371/journal.pone.0064030

**Published:** 2013-05-15

**Authors:** Hwajin Yang, Sujin Yang, Giho Park

**Affiliations:** 1 Psychology, School of Social Sciences, Singapore Management University, Singapore; 2 School of Medicine, The Catholic University of Korea, Seoul, South Korea; 3 Singapore-MIT Alliance for Research and Technology, Singapore; Universidade Federal do ABC, Brazil

## Abstract

The literature on directed forgetting has employed exclusively visual words. Thus, the potentially interesting aspects of a spoken utterance, which include not only vocal cues (e.g., *prosody*) but also the *speaker* and the *listener*, have been neglected. This study demonstrates that prosody alone does not influence directed-forgetting effects, while the sex of the speaker and the listener significantly modulate directed-forgetting effects for spoken utterances. Specifically, forgetting costs were attenuated for female-spoken items compared to male-spoken items, and forgetting benefits were eliminated among female listeners but not among male listeners. These results suggest that information conveyed in a female voice draws attention to its distinct perceptual attributes, thus interfering with retention of the semantic meaning, while female listeners' superior capacity for processing the surface features of spoken utterances may predispose them to spontaneously employ adaptive strategies to retain content information despite distraction by perceptual features. Our findings underscore the importance of sex differences when processing spoken messages in directed forgetting.

## Introduction

A key characteristic of adaptive memory processing is forgetting information that is no longer needed. Forgetting is empirically important for both healthy memory function and effective learning processes in both normal and clinical populations, because it helps individuals overcome unwanted or unpleasant memories of past events and enhances learning and remembering by optimizing both the encoding and retrieval of information [1]. Directed (or intentional) forgetting refers to the purposeful loss of information that has been successfully encoded but designated as unimportant. Studies of this phenomenon have widely used a procedure in which participants are presented with two lists of words (List 1, List 2) and instructed to either remember or forget those lists (for a review, see [2]). The “remember” group is instructed to remember both List 1 and List 2, but the “forget” group is told to forget List 1 and to retain only List 2. In a later test, these groups are asked to recall as many words as possible from both lists. Research using this paradigm has typically shown two robust effects: (1) *forgetting costs*, which refer to the impaired recall of List 1 in the forget group relative to the remember group, and (2) *forgetting benefits*, which refer to the enhanced recall of List 2 in the forget group relative to the remember group.

Several theories account for these effects. The *retrieval-inhibition* theory proposes that forgetting costs occur because the forget instruction suppresses access to List 1 items, whereas benefits occur due to the forget group's escape from proactive interference [3]. The *selective-rehearsal* theory assumes that costs and benefits occur because the forget instruction facilitates selective rehearsal of List 2 at the expense of List 1 [4]. Recent research, however, proposes that different mechanisms underlie forgetting costs and benefits. For instance, the *context-strategy* theory attributes the costs to a mismatch between the encoding context and the testing context of List 1 items, and the benefits to better study strategies during List 2 learning [5]. The *reset-of-encoding hypothesis*, in contrast, attributes the costs to retrieval inhibition and the benefits of a reset of encoding processes that facilitate the encoding of List 2 items as effectively as the encoding of List 1 items [6].

Our purpose for the study was twofold. First, we aimed to investigate the influence of emotional prosody – e.g., an angry voice – on directed forgetting. In everyday communication, specific and discrete emotional states are frequently manifested not only in the content of the spoken word but also in recognizable nonverbal cues such as prosody, which refers to the vocal expression of emotions through pitch contour, intensity, or duration [7]. Moreover, prosody often reflects the presence of emphasis or contrast by which a listener is able to understand the intended meaning when prosodic information is correctly retrieved. One example of this phenomenon is sarcasm, in which the speaker uses tone of voice to display a dissociative attitude. Thus, the efficient scrutiny of a specific acoustic profile of those prosodic parameters is useful for decoding emotional content [8]. While directed-forgetting studies to date have not systematically investigated the effect of emotional prosody, previous directed-forgetting studies on emotional valence, although somewhat mixed [9], suggest that emotional material is relatively resistant to forgetting. For instance, a robust resistance to directed forgetting was reported for emotional pictures [10], threat-related words [11], and negative memories [12]. It is uncertain, however, whether emotional prosody would have the same effect on directed forgetting.

Second, we sought to investigate how the sex of the speaker and the sex of the listener influence directed forgetting. Spoken messages reflect various features of the speaker, and their influence is interdependent on the interaction with the listener [13]. Evidence collected from adult speakers and listeners suggests that sex differences are an important and systematic source of acoustic variation in both the perception and production aspects of speech and language (for a review, see [14]). For instance, in terms of perceptual aspects as *listeners*, women distinguish prosodic information more quickly than men [15] and make use of such information sooner during word processing [16]. Women – but not men – also integrate prosodic attributes into word processing even when it is not relevant to the task [15] and process prosodic information preattentively when prosodic attributes are unattended [16]. Thus, women's enhanced sensitivity to prosodic information may allow women listeners to make use of different forgetting (or remembering) processes for emotionally spoken utterances than those used by men.

In terms of production aspects as *speakers*, on the other hand, it is important to note that males and females have anatomically different sizes and shapes of the vocal tract, which filters sound that is produced at the sound source, the larynx [17]. As a result, a wealth of evidence documents clear sex differences across wide-ranging acoustic measurements that represent articulatory differences. For example, women display higher fundamental frequency (F0, which determines the pitch of a voice) and spectral formant frequencies (F1, F2, F3, and F4, which determine the perceived timber of a voice), but lower formant amplitude (which determines vocal-intensity level) than men (for a review, see [18]). Moreover, women use more vocal jitter (fundamental frequency perturbation) but less vocal shimmer (amplitude perturbation) than do men. Additionally, women typically make greater use of pitch and manipulate inflection to emphasize points, whereas men do not use their highest level of pitch but control volume instead [19]. Noting these apparent sex differences in vocal attributes, it seems plausible that the female voice that is expressed with seemingly distinct acoustic parameters – e.g., a higher pitch level, wider pitch range, or a greater vocal jitter – could be perceived as more salient than and easily differentiated from the male voice [20]. In line with this idea, developmental studies demonstrate that infants typically show significant preference for speech featured with higher pitch, broader pitch range, and faster tempo, i.e., mother's voice [21]. More direct evidence comes from brain-imaging studies. Lattner, Meyer, and Friederici [22] first investigated brain activation in response to male and female voices and found that the activation pattern was stronger in response to the female voice. The authors contend that this effect could be because (a) a female voice is perceptually more salient than the male voice or (b) a female's high-pitched voice signals her increasing stress, which should alert the listener to potential hazards or social tension. Given that these acoustic properties become an integral part of the perceptual record in memory [23], [24], it is thus possible that sex differences in both the perception and production aspects of language may have different effects on the process of forgetting and remembering [25]. Hence, studying the sex of the speaker and the listener in the context of directed forgetting is critical.

### The Present Experiment

Adults can identify angry prosody with greater precision than other emotional prosody, such as fear, disgust, or joy [26], becausee angry prosody is typically characterized by its distinctive temporal structure, amplitude (loudness), roughness, and pitch [8]. Thus, we decided to focus on angry prosody. The content of the spoken word was manipulated to be neutral so that a clear distinction could be drawn between the semantic content of a word and the prosody of its utterance, especially when semantic content and prosody are independent.

Past research has demonstrated that neutral words spoken with emotional prosody and emotional words alike capture attention more readily than those spoken with neutral prosody, suggesting that emotional prosody may involve cognitive outcomes similar to those resulting from emotional words (for a review, see [27], [28]). Research using event-related brain potentials (ERPs), however, has shown that the neurocognitive mechanisms for processing information from emotional semantic cues versus emotional prosody are dissociable, indicating that emotional semantics and emotional prosody in speech may be treated differently [29]. Consistent with this suggestion, behavioral research on spoken-language processing suggests that while vocal features in a spoken word are retained in episodic memory [30]–[32], their impact on the retention of the semantic content is not significant. For instance, Schirmer [7], [33]–[34] has recently found that emotional prosody alone does not enhance memory storage of the word's meaning. These findings suggest that emotional prosody may not necessarily result in beneficial effects on memory of semantic content – as opposed to emotional words, which have typically shown memory enhancement (for a review, see [33]). We propose two causes. First, this may be because perceptually salient prosodic attributes (e.g., wide-ranging pitch, timber, or volume) capture attention readily but subsequently divert cognitive resources and encoding effort from learning the *content*. Consistent with this, the literature demonstrates that although emotional prosody does not facilitate memory, it alters affective representation of the words in memory [7], [30]–[32]. That is, participants are more prone to rate neutral words presented with emotional prosody (either sad or happy) as more emotional (either negatively or positively) than those with neutral prosody. This suggests that attention capture by emotional prosody heightens percept-based representation in memory rather than meaning-based encoding, which in turn is likely to divert cognitive resources away from encoding the content.

Secondly, we propose that the match between emotional valence and word meaning may result in different encoding processes. It is noteworthy that our stimuli – neutral words spoken with emotional prosody (e.g., “pencil” spoken angrily) – are distinguished from emotionally charged words or pictures (e.g., “snake”). Specifically, the emotional valence of angry prosody (negative) is not congruent with the neutral meaning of the word, while the valence of emotion-laden stimuli (e.g., negative) is congruent with its emotional semantics. Given that evaluation of such incongruence between perceptual valence and semantic content would typically require more cognitive resources (such as cognitive processing time) for encoding [35], we can assume that attention capture by emotional prosody may not be beneficial for encoding the semantic content of the word. In contrast, attention capture by emotion-laden stimuli may be conducive to encoding the emotional content of the word because the congruence between its emotional valence and semantic content helps to facilitate encoding processes. This accounts for why emotionally charged stimuli (e.g., “snake”) enhance memory, but the emotional prosody of neutral words does not. Such encoding benefits for emotionally charged stimuli also explain why those stimuli are resistant to directed forgetting [10]–[12]. Given this, our hypothesis is that the emotional prosody of neutral words would not affect the forget group, since encoding (or rehearsal) effort is unnecessary for forgetting; it would, however, hinder encoding (or rehearsal) effort in the remember group, primarily due to the attention drawn to prosodic information that is incongruent with the semantic content.

Regarding the effect of the sex of the speaker, we hypothesized that the sex of the speaker would modulate directed forgetting for a spoken utterance. In view of apparent sex differences in the productive aspects of spoken messages, we expected that a female voice, compared to a male voice, would promote perceptual encoding rather than semantic encoding because of more salient acoustic properties of a female voice than those of a male voice. Given the literature that has found that pitch, among other acoustic parameters, makes a significant contribution to perceptual discrimination of sounds [36], it is plausible that a female voice – which is typically characterized by high and wide-ranging pitch – would draw attention primarily to perceptual attributes, as readily as prosodic attributes. It should be noted, however, that the sex of the voice (male voice vs. female voice) is independent of valence. Namely, in contrast to neutral words spoken with angry prosody, those spoken in either a male or a female voice do not necessarily involve incongruent information between the perception of the speaker's voice and their neutral meaning. It is therefore possible that although female voice and angry prosody alike are perceived as salient, their impacts on memory (i.e., forgetting and remembering) can differ to an extent, depending on information congruency between perceptual valence and emotional semantics. Given that perceptual and semantic incongruence would typically usurp cognitive resources (such as cognitive processing time) from encoding and rehearsal of the content, we expect that the sex of the speaker – which does not comprise incongruence information – impairs remembering (as opposed to forgetting) to a lesser degree than does emotional prosody.

On the other hand, we expect that female listeners would take advantage of prosodic cues by adopting more adaptive strategies (e.g., effective encoding, selective rehearsal) because of female listeners' greater sensitivity to nonverbal cues (e.g., [16]). Consistent with this view, Wilding and Cook [37] demonstrated that females were able to recognize the speaker's voice even after a one-week retention interval but males were not, suggesting that females outperform males in voice recognition. In prior studies, females were also found to outperform males (a) in short-term memory tasks that involve learning lists of words [38]–[40], (b) in tasks to remember phonologically familiar *novel* words [41], (c) in verbal episodic-memory tasks requiring verbal processing (for a review, see [42]), and (d) even in foreign language learning [43]. This line of evidence suggests that females may be more resistant to forgetting and better at remembering due to their advantages in verbal memory. Accordingly, we expect that the sex of the listener would influence directed-forgetting processes via changes in either forgetting – for example, via better retrieval – or remembering – such as via better strategies to deal with proactive interference.

Taken together, our predictions that the directed-forgetting effect would be moderated by either the prosody or the sex of the speaker and the listener can be tested by higher-order interactions among the study list (List 1 and List 2), memory instruction (forget, remember), prosody (neutral, angry), and the sex of the speaker or the listener. It should be noted, however, that as this study is the first of its type – and preliminary – we do not endorse specific hypotheses pertaining to how forgetting costs and benefits would be influenced by the specific combination of the prosody (neutral, angry), the sex of the speaker, and the sex of the listener.

## Method

### Participants

Participants were 165 undergraduate students. Eighty-one participants were assigned to the forget group (*N*_male_ = 41) and 84 (*N*_male_ = 42) to the remember group. All participants reported normal or corrected hearing. They gave signed informed consent prior to the experiment. All procedures were reviewed and approved by the Institutional Review Board at Singapore Management University.

### Design

We used a LIST (List 1, List 2) x CUE (forget, remember) x PROSODY (neutral, angry) x SPEAKER (female voice, male voice) x LISTENER (female, male) mixed-factor design, with CUE and LISTENER as between-participant factors and the remainder as within-participant factors.

### Materials

Male and female actors produced 325 voice samples in either a neutral or angry tone. These vocal samples were digitally recorded at a 16 bit/44.1 KHz sampling rate, with the amplitude normalized at the root-mean-square value. Twelve lay listeners heard these words over a headset and were asked to type them on a computer keyboard, and words that were accurately identified by all 12 listeners were selected for subsequent ratings. A group of 30 independent raters used a 5-point scale to rate visually presented words for word valence and word arousal. After this, raters were auditorily presented with words and asked to identify the gender and prosody of each vocalization and to rate them on a 5-point scale for emotional valence, emotional arousal, and intensity of angriness. Praat software was then used to extract several acoustic parameters of the selected words: duration, pitch (F0), intensity, and spectral formants (F1, F2, F3, F4). A total of 32 disyllabic nouns that had neutral valence and were weakly arousing were selected for the study and divided into two lists of 16 neutral items each for counterbalancing purposes (see the Appendix S1 for the entire set). The two lists were approximately matched on mean word length (List_1_ = 5.8, List_2_ = 6.0), word frequency (Kucera-Francis Written Frequency: List_1_ = 54.6, List_2_ = 62.3), word valence, word arousal, emotional valence, emotional arousal, and emotional intensity (Table 1). Acoustic analyses using Praat (Table 2) ensured that male-spoken items significantly differed from female-spoken items, particularly in the third and fourth formants (F3, F4). These are most salient acoustic features in the gender classification of natural voices, because they depend on the shape of the pharyngeal cavity, which is disproportionably larger in males [44]. In addition, angrily spoken items significantly differed from neutrally spoken items in pitch, intensity, and the first formant (F1). Each list consisted of an equal number of angry-prosody and neutral-prosody items, half spoken by a male voice and the other half by a female voice.

**Table 1:** Stimulus rating results.

	Neutral Prosody			Angry Prosody		
	List 1	List 2	*t*	List 1	List 2	*t*
Word valence*^a^*	3.2 (.19)	3.0 (.24)	1.8	3.15 (.33)	2.88 (.23)	1.9
Word arousal*^b^*	2.1 (.47)	2.0 (.45)	.95	2.2 (.41)	2.2 (.32)	.09
Emotional valence*^a^*	3.1 (.11)	3.0 (.14)	1.8	1.98 (.33)	2.03 (.34)	−.32
Emotional arousal*^b^*	1.6 (.14)	1.5 (.17)	1.8	2.54(.23)	2.5 (.23)	.32
Intensity of angriness*^c^*	1.05 (.04)	1.06 (.06)	.54	3.3 (.56)	3.2 (.7)	.30
Gender identification accuracy (%)	99	99	.28	97.5	98.7	−.72
Tone identification Accuracy (%)	99	99	.01	89	90	−.54

*Note.* Standard deviations are displayed in parentheses. The two lists were not significantly different in any of these psychological properties. Ratings were based on a 5-point Likert scale. Word valence and arousal were assessed for visually presented words, whereas emotional valence and arousal were assessed for vocal samples. aValence was rated on a scale from 1 (*very negative*) to 5 (*very positive*). bArousal was rated on a scale from 1 *(non-arousing)* to 5 *(very arousing)*. cIntensity of angriness was rated on a scale from 1(*not at all angry*) to 5 (*very angry*).

**Table 2:** Acoustic parameters of voice samples.

	Neutral Prosody			Angry Prosody					
	Male	Female	*t*		Male		Female		*t*
Duration (ms)	584 (90)	599 (60)	−.38			638 (85)		527 (97)	2.4^*^
Mean Pitch (F0, Hz)	135 (31)	213 (7.4)	−6.8^***^			268 (22)		283 (22)	−1.4
Max. Pitch	195 (93)	275 (73)	−1.9			343 (24)		348 (31)	−.32
Min. Pitch	103 (30)	169 (22)	−4.9^***^			177 (41)		188 (26)	−.63
Mean Intensity (dB)	68 (2.4)	70 (1.6)	−1.8			73 (2.2)		66 (2)	6.9^***^
Max. Intensity	74 (2.3)	74 (1.5)	−.26			79 (1.9)		73 (1.8)	6.2^***^
Min. Intensity	49 (5.3)	54 (6.9)	−1.54			46 (8.9)		45 (7.4)	.21
1^st^ Formant (F1, Hz)	609 (91)	552 (123)	1.07			801 (126)		681 (105)	2.1^†^
2^nd^ Formant (F2, Hz)	1641 (245)	1834 (376)	−1.22			1876 (185)		1901 (378)	−.17
3^rd^ Formant (F3, Hz)	2869 (125)	3085 (189)	−2.7^*^			2892 (133)		3056 (185)	−2.04^†^
4^th^ Formant (F4, Hz)	3963 (208)	4333 (154)	−4.04^**^			3980 (151)		4239 (92)	−4.1^**^

*Note*. Standard deviations are displayed in parentheses. (†<.08. *p<.05. **p<.01. ***p<.001).

### Procedure

Before participants began the main task, they were asked to rate their current mood state on a 9-point Likert scale that ranged from 1 (*very bad*) to 9 (*very good*), with a response of 5 indicating neutral mood. After this, the main experiment began, following the typical directed-forgetting paradigm. Participants heard two lists of 16 words at a rate of 5 sec per item, including an inter-stimulus interval. Participants in the forget group first studied List 1, but were then told that List 1 was only for practice to familiarize them with the task. They were also told that their memory for List 1 would not be tested and were encouraged to forget the list. The remember group, however, was told to keep remembering the items for a later memory test, because the list they had studied was only the first half of the complete list. Thus, the instruction explicitly specified that participants should either forget or remember the first list. Participants in both groups then studied List 2 in the same fashion and were told to remember the items for a later test. The final-recall test was preceded by a 90 sec filler task (a simple math task). Participants then recalled as many items as possible from both lists and in any order. After the recall task had been completed, all participants were asked to rate four mood states (*pleasantness, tension, tiredness, and anxiousness*) on Likert scales that ranged from −5 (*very unpleasant; very tense; very tired; very anxious*) to +5 (*very pleasant; very relaxed; very energetic; very calm*). These mood measures served to rule out the possibility that experienced mood states could affect directed forgetting [45]. When a participant had completed the survey, he or she was fully debriefed as to the purpose and hypothesis of the experiment and thanked for their participation.

## Results and Discussion

**Figure 1:**
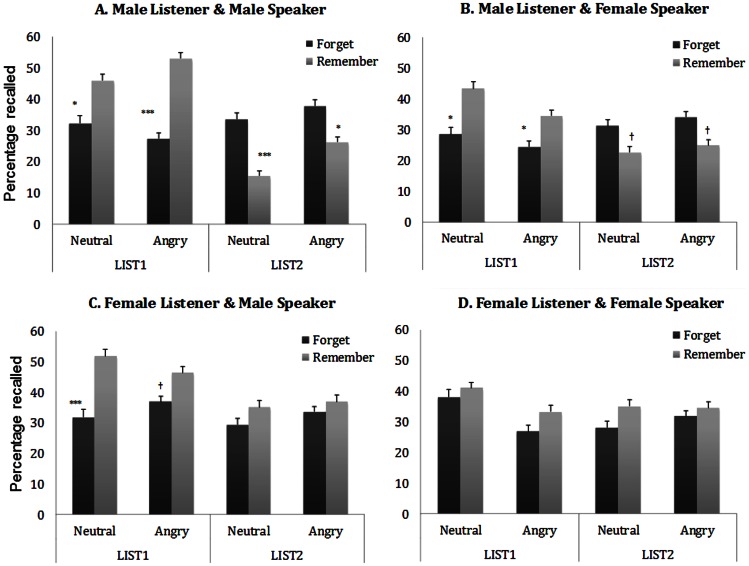
Proportion of items recalled as a function of list (List 1, List 2), emotional prosody (Neutral, Angry) and cue (Remember, Forget). (a) illustrates the results of male listeners for items spoken by a male voice. (b) illustrates the results of male listeners for items spoken by a female voice. (c) illustrates the results of female listeners for items spoken by a male voice. (d) illustrates the results of female listeners for items spoken by a female voice. Error bars represent the standard error. (†<.08. *p<.05. **p<.01. ***p<.001).

Overall recall rates (Figure 1) were analyzed globally, with a LIST (List 1, List 2) x CUE (forget, remember) x PROSODY (neutral, angry) x SPEAKER (female, male) x LISTENER (female, male) mixed-factor ANOVA, for theoretically important effects. Consistent with the literature, significant directed-forgetting effects were captured by the LIST x CUE interaction, *F*(1, 161) = 33.3, *p*<.001, η2 = .15. Notably, the LIST x CUE interaction (i.e., the directed-forgetting effect) was not qualified by PROSODY, *p*>.9, suggesting that prosody did not affect directed forgetting. We found, however, that the LIST x CUE interaction was qualified by the sex of either the speaker or the listener, as indicated by three-way interactions with SPEAKER, *F*(1, 161) = 5.7, *p* = .018, η2 = .03, and LISTENER, *F*(1, 161) = 5.5, *p* = .02, η2 = .08. We will discuss these results below in greater detail. As customary with previous studies, separate results from the analyses of the costs (i.e., forgetting costs for List 1) and the benefits (i.e., forgetting benefits for List 2) are presented and discussed below.

### Forgetting Costs for List 1

A CUE x PROSODY x SPEAKER x LISTENER mixed-factor ANOVA was performed on the List 1 recall rates. The main effect of PROSODY was that List 1 items were recalled better when spoken neutrally than angrily, *F*(1, 161) = 4.12, *p* = .044, η^2^ = .03, suggesting that when compared to neutral prosody, angry prosody impaired memory of semantic content. As expected, a significant interaction between CUE and SPEAKER was observed, *F*(1, 161) = 6.15, *p* = .014, η^2^ = .03. Planned comparisons indicated that forgetting costs were less pronounced when items were spoken by a female voice, *t*(163) = −2.7, *p* = .01, than a male voice, *t*(163) = −5.4, *p*<.001. Follow-up analysis of this interaction indicated that relatively attenuated costs for female-spoken items were attributable to the remember group. That is, a reduced group difference – which underlies forgetting costs – for female-spoken items was due to the remember group who recalled female-spoken items substantially less (*M* = 38.1%) than male-spoken items (*M* = 49.4%), *t*(83) = −4.25, *p*<.001. Additionally, we found an interaction between SPEAKER and PROSODY, indicating that this significantly lower recall for female-spoken items was more pronounced when items were spoken angrily than neutrally. This suggests that female-spoken items interfered with memory processing, especially when spoken with angry prosody. Finally, we found a four-way interaction between SPEAKER, PROSODY, CUE, and LISTENER, *F*(1, 161) = 4.68, *p* = .03, η^2^ = .03. This four-way interaction was difficult to interpret, but it appears to indicate that the significantly lower recall for items spoken by a female's angry prosody was more pronounced among male listeners in the remember group than their female counterparts.

Our key findings are summarized as follows. First, impaired recall for the semantic content of a word (i.e., greater forgetting) was more pronounced when items were spoken by a female voice. Second, such impairment in recall performance was more apparent when female speakers used angry prosody than neutral prosody. Third, memory interference caused by perceptual attributes of the spoken utterance was more evident for the remember group – whose participants were instructed to remember the list – than for the forget group. And fourth, male listeners' recall was poorer than female listeners'. These findings are, in part, consistent with our expectation that drawing attention to perceptually salient attributes of the spoken word would interfere with encoding and subsequent rehearsal, thereby making recall more difficult.

### Forgetting Benefits for List 2

When the same ANOVA analysis was performed on the List 2 recall rates, the main effect of PROSODY for List 2 was still observed, *F*(1, 161) = 4.67, *p* = .03, η^2^ = .03, but the direction of its effect was contrary to the one observed for List 1. Specifically, the enhanced recall of List 2 was obtained for angrily spoken items, whereas the enhanced recall of List 1 was obtained for neutrally spoken items. This suggests that the impact of emotional prosody on memory may be constrained by temporal variables such as retention interval or time delay. This issue will be discussed further in the following section. Notably, we found a significant CUE x LISTENER interaction, *F*(1, 161) = 9.62, *p* = .002, η^2^ = .05, indicating that forgetting benefits were qualified by the sex of the listener. Follow-up analysis showed that the benefits were still observed among male listeners, *t*(81) = 3.5, *p* = .001, but disappeared among female listeners, *p* = .27, who showed a small group difference between the remember and the forget conditions. Further analyses demonstrated that female listeners outperformed male listeners in the remember condition, *t*(82) = 3.54, *p* = .001, but not in the forget condition, *t*(79) = −.87, *p* = .39. Thus, disrupted benefits among female listeners were attributable to females' enhanced recall in the remember condition compared to the forget condition. This suggests that female listeners in the remember group may employ strategies to suppress interference with previous List 1 learning. No other effects were significant.

### Gender Effects on Directed Forgetting

Because we found evidence that directed-forgetting effects were qualified by either SPEAKER or LISTENER, we examined more specifically how sex differences might delimit forgetting costs and benefits. To this end, we performed multiple LIST x CUE x PROSODY mixed-factor ANOVAs within each of the subgroups, which were created according to the sex of the speaker and listener. We describe significant results that bear directly on the current purpose. When men heard a male voice (Figure 1a), the LIST x CUE interaction – which implies the typical directed-forgetting effect – was significant, *F*(1, 81) = .51, *p*<.001, η^2^ = .33, and this effect was not qualified by PROSODY, *F*(1, 81) = .39, *p*>.53. Follow-up tests of the LIST x CUE interaction revealed that both costs and benefits were significant, *p*s<.001. This indicates that regardless of the prosody, fewer items were recalled from List 1 in the forget group than in the remember group, while more items from List 2 were recalled in the forget group than in the remember group. When men heard a female voice (Figure 1b), a significant directed-forgetting effect was still found, *F*(1, 81) = 18.2, *p*<.001, η^2^ = .17. Again, this effect was not qualified by PROSODY, *p*>.6. Follow-up tests of this interaction revealed significant costs, *ps*<.05, but marginal benefits, *ps*<.08. When women heard a male voice (Figure 1c), the LIST x CUE interaction was marginally significant, *F*(1, 80) = *3.7, p = .*058, η^2^ *= .*04, without being qualified by PROSODY, *p*>.4. Follow-up analysis of this interaction showed significant costs, *t*(80) = −3.10, *p* = .003, but no benefits because of women's enhanced recall in the remember condition compared to the forget condition, *p*>.29. Finally, when women heard a female voice (Figure 1d), the LIST x CUE effect was neither significant, *p*>.9, nor qualified by PROSODY, *p*>.37. Follow-up analysis revealed neither the costs nor the benefits, *p*s>.3.

### Analysis of List Output Order

We noted that gender effects on directed forgetting were attributable to the remember condition. This raises the possibility that sex differences in directed forgetting may have been due to sex differences in the output order of the two lists in the remember condition. For instance, given that list order affects recall performance [6], females' superior List 2 recall can be observed when females in the remember group recall List 2 before List 1, whereas their male counterparts recall List 1 before List 2. Thus, we investigated whether differential enhancement for List 2 could be accounted for by sex differences in preferred output order in the remember condition. Although we did not instruct participants to recall words in a given order, some participants recalled spontaneously in list-based chunks, i.e., recalling most of items from one list first and then from the other. Depending on the first list that was recalled, participants were grouped into either List 1 (*n* = 68) or List 2 (*n* = 42). Our grouping criteria allowed very little intrusion – at the most, one item from the other list. Those who recalled items in a mixed-list pattern (with two or more items intruding from the other list) were classified with the mixed-list group (*n* = 55). Three types of list-order analyses were performed, as described below.

First, a chi-square test revealed no association between output preference (L1, L2, Mixed) and the sex of the listener (male, female), χ2(2) = .11, *p* = .96, suggesting that the list-output order (hereafter, called LIST ORDER) was independent of the sex of the listener. Second, we performed a repeated-measures mixed factor ANOVA by LIST x CUE x PROSODY x SPEAKER x LISTENER x LIST ORDER. Results showed neither the main effect of LIST ORDER, *F*(2, 153) = .38, *p* = .68, nor two-way interactions between LIST ORDER and PROSODY, SPEAKER, and LISTENER, respectively, *p*s >.19. More importantly, the three-way interaction between LIST ORDER, LIST, and CUE was not significant either, indicating that the directed-forgetting effect (as indicated by the LIST x CUE interaction) was not qualified by LIST ORDER, *F*(2, 153) = .20, *p* = .82. Finally, we examined whether List 2 benefits, which were only evident in male listeners, could be due to male listeners' list-order preference. When the List 2 recall rates of male participants were entered into a mixed-factor ANOVA by CUE x PROSODY x SPEAKER x LIST ORDER, the interaction between CUE and LIST ORDER was not significant, indicating that the List 2 benefits observed in males were not influenced by the list-output order, *F*(2, 77) = .103, *p* = .90. Taken together, these results suggest that sex differences in directed forgetting, at least in our study, are not attributable to sex differences in the output order of the lists.

### Self-reported Pre-task and Post-task Mood Ratings

An independent-samples *t*-test performed on pre-task mood ratings revealed no difference between the remember group and the forget group, *p*>.9. A series of independent-samples *t*-tests was performed to determine any post-task mood differences between the groups on four mood items (*pleasantness, tension, tiredness, and anxiousness*). None of those items revealed significant group differences (Table 3), all *p*s>.23. These results rule out the possibility that listeners' emotional states affected directed forgetting.

**Table 3:** Mood Measures as a Function of the Instruction Cue and the Sex.

		Post-task mood^b^				
	Pre-task mood^a^	Unpleasant– Pleasant	Tensed – Relaxed	Tired – Energetic	Anxious – Calm	
Remember (*n* = 84)	5.4 (1.8)	.39 (1.9)	.44 (2.2)	−.52 (2.3)		.61(1.9)
Forget (*n* = 81)	5.4 (1.6)	.30 (1.7)	−.06 (1.8)	−.46 (1.9)		.37 (1.7)
*p*	.91	.75	.23	.85		.42
Female (*n* = 81)	5.5 (1.7)	.22 (1.7)	−.02 (1.99)	−.95 (1.9)		.30 (1.9)
Male (*n* = 84)	5.9 (1.6)	.47 (1.9)	.53 (2.04)	−.05 (2.1)		.77 (1.8)
*P*	.19	.39	.08	.006		.20

*Note. SD*s are shown in parentheses. The *p* represents a test of the significance of the difference between the two groups.

a Pre-task mood was examined on a 9-point Likert scale anchored between 1 and 9, with a response of 5 indicating a neutral state.

b Post-task mood was examined on a 11-point Likert scale anchored between −5 and +5, with a response of 0 indicating a neutral state.

Similar analyses were performed to examine any sex differences in self-reported mood states (Table 3). There was no sex difference in pre-task mood ratings, *p*>.19. A significant sex difference, however, was found in the degree of tiredness, *t*(162) = −2.8, *p* = .006, indicating that female participants felt significantly more tired than male counterparts when they had completed the memory task. Given that there was no sex difference in pre-task mood at the outset of the experiment, this post-task mood difference in tiredness could have occurred due to different effort levels put forth by each sex. This result implies that female listeners might have tried harder or exerted more energy than male listeners in learning the word lists.

## General Discussion

Our findings demonstrate that the sex of the speaker and the listener modulate directed-forgetting effects. Forgetting costs for List 1 were robust for male-spoken items but attenuated for female-spoken items. Forgetting benefits were still evident among males, but eliminated among females. Prosody did not modulate directed-forgetting effects.

It is notable that attenuated costs for List 1 items spoken by a female voice were induced by poorer recall in the remember group than the forget group. We would argue that these impaired costs occurred because the attention drawn to perceptually distinct voice attributes usurped substantial processing resources, thereby decreasing the effort available to encode and rehearse the meaning of the material. This suggests that voice attributes and the semantic meaning of the spoken utterance may be processed in parallel, thereby competing with each other for cognitive resources. Additionally, the fact that poorer recall for female-spoken items was more pronounced when they were spoken with angry prosody than neutral prosody suggests that although emotional prosody alone does not significantly affect directed forgetting, emotional prosody spoken by a female voice renders its utterance more salient and modulates subsequent memory processes. Taken together, these results suggest that perceptually salient vocal features may hinder intentional remembering (but not forgetting) of the content message.

Extant theories do not readily account for our finding that forgetting costs were induced by poorer performance in the remember condition than in the forget condition, because they postulate that forgetting costs are due to decreased recall in the forget group relative to the remember group. Given this, our finding raises both theoretically and empirically important questions as to whether impaired costs due to a remember condition can still be regarded as such. To date, studies of directed forgetting have centered on variables that could modulate memory processing in the forget condition. For example, emotionally charged words are difficult to suppress, even given the intention to forget them [10]. Positive mood also eliminates forgetting costs due to associative activation of List 1 items during List 2 learning [43]. These studies have reported disrupted forgetting costs caused by an increased recall of List 1 in the forget group, suggesting that emotional valence and experienced mood undermine forgetting. Our study, however, is the first to reveal an important factor that affects the remember condition without affecting the forget condition, suggesting that attentional bias to salient physical attributes and a subsequent reduction in cognitive resources impair intentional remembering. This appears to contradict the literature, which suggests that the emotional valence of the stimulus (either words or pictures) captures attention readily and renders the event more persistent in memory and resistant to forgetting [10]–[11], [45]. It should be noted, however, that there is a major difference between our findings and the literature: We manipulated perceptual features of the spoken message independent of its semantic content, which was controlled to be neutral. By contrast, the majority of studies have directly manipulated the semantic content of the message to be emotionally significant without changing perceptual attributes. Therefore, our results neither contradict nor disprove previous findings.

Forgetting benefits for List 2, on the other hand, were still evident among male listeners but eliminated among female listeners. It is noteworthy that this effect was attributable to a decrease in group differences induced by the enhanced recall of female listeners in the remember condition. We would argue that the absence of benefits in female listeners occurred because they adopted progressively better encoding strategies for List 2 items to suppress interference accrued from List 1 learning. Noting females' enhanced sensitivity to prosodic information and superior retention of the speaker's voice or verbal material [7], [33], it is plausible that females are likely to take advantage of physical features of the spoken utterance and to encode and retain the surface features of the spoken stimulus. Moreover, there is some evidence suggesting females' use of better strategies for List 2 items. First, we found that female listeners in the remember condition showed significantly greater recall for List 2 (*M* = 35.4%) than their male counterparts (*M* = 22.3%). Given that List 2 encoding followed List 1 encoding, the superior recall of females, despite high memory load, could be attributable to effective strategies for List 2 learning. This pattern, however, was not observed in the forget condition, in which females were not required to remember List 1 items, and thus the perceived need to employ strategies was not evident. Second, given that encoding voice information requires cognitively effortful processes [46], sex differences in post-task tiredness suggest that females expended more effort than males to remember List 2 items, which should entail mnemonic strategies based on vocal features. And third, an interesting parallel was observed in recall performance between female listeners in our study and participants in Sahakyan and Delaney's study [47], who were required to employ deeper encoding of List 2 items. Taken together, these results suggest that the elimination of forgetting benefits among female listeners is due to their active use of encoding strategies.

It is worth noting that contrary to our expectations, prosody alone was irrelevant for directed forgetting, which suggests that emotional prosody does not necessarily result in cognitive outcomes similar to those of emotional words [10]. Given recent empirical studies that have demonstrated that recognition memory was comparable for both neutrally and emotionally spoken words [7], [34], this failure of emotional prosody is not surprising. It is, however, important to note that when separate analyses were performed with respect to List 1 and List 2 recall rates, the enhanced recall of List 2 was for angrily spoken items, whereas the enhanced recall of List 1 was for neutrally spoken items. This suggests that the impact of emotional prosody on memory may be constrained by temporal variables such as retention interval. According to the literature proposing two possible modes of memory operation for sounds [48], memory for sound stimuli can be formed either through the trace mode, which is based on the sensation produced by sound stimuli, or the context-coding mode, which is based on the meaning of sound. The efficiency of these modes is known to depend on the retention interval between encoding and retrieval. For instance, with a short retention interval, the trace mode enhances memory for sound with perceptually salient attributes, whereas with a long retention interval, the context-coding mode enhances memory for sound whose semantics are well encoded and represented. Our findings are consistent with this theoretical and empirical view, since memory for recently presented List 2 items was greater for perceptually more salient angry-prosody items, while memory for List 1, which was temporally more distant, was greater for neutral-prosody items, which did not distract attention from their meanings and thus were likely to lead to semantic coding.

Moreover, our finding that memory for female-spoken items was poorer when spoken with angry prosody than neutral prosody indicates that emotional prosody expressed by a female voice appears to make an utterance more salient, thus resulting in attentional focusing on perceptual features instead of its content message. Given that the female voice is typically characterized by acoustic parameters such as higher pitch level, wider pitch range, or a greater vocal jitter – all of which are likely to cause a female voice to be perceived as lighter and less aggressive (and thus more noticeable) than a male voice [49] – this finding suggests the importance of contextual factors that can potentially modulate the effect of emotional prosody on memory. Since our study was limited to a single word with no context, it is thus important that future studies examine how the perceptual salience of emotional speech affects memory processing. For instance, noting that a speech stream (e.g., phrases or a short sentence) spoken with emotional prosody can be perceived as more salient than a single word in isolation from its context, it will be interesting to study the effect of emotional prosody on memory with speech stimuli that engage complex vocal attributes (e.g., inflection) and, in turn, heighten the perceptual salience of prosodic information.

Our analyses of both pre-task and post-task mood data further suggest that memory for vocal emotional expressions was independent of experienced mood states during the study, implying that the prosody effect (i.e., a female's angry voice) would result from differences in the focus of attention rather than from changes in mood. Taken together, these results suggest that perceptually distinctive vocal features may hinder intentional remembering (but not forgetting) of the content message.

We note our caveat of having only four study items for each PROSODY X SPEAKER condition, but there is little chance that our effects are spurious: They emerged from a well-controlled laboratory experiment, with a sample size adequate for the number of explanatory variables; the observed magnitude of our effect sizes indicates statistically meaningful relationships; and our results are based on confirmatory analyses rather than an exploratory analysis (for a review, see [50]). Moreover, given that our voice samples were digitally recorded and rigorously selected after pretesting, our findings cannot be attributed to any systematic errors associated with the voice stimulus.

We also note that deficits in semantic processing for items spoken by a female voice could be due in part to differing methodologies, including various aspects of design and implementation. For instance, it is possible that the distinctiveness of female-spoken items over male-spoken items could be heightened by the intermixed presentation of items. Although we believe that mixed presentation with both neutral and angry prosody emulates real-life social interactions and communication better than a blocked presentation, future studies are warranted to clarify whether memory deficits for female-spoken utterances can be affected by other aspects of the design or implementation. Given that our study provides the first evidence of its kind, more studies are warranted to examine the operative mechanisms of gender-modulating effects on directed forgetting.

In conclusion, our key finding suggests that attentional bias to peripheral and perceptually salient vocal attributes interferes with intentional remembering of the semantic content, rendering the information less enduring or accessible for subsequent retrieval. Specifically, in contrast to emotional valence – which has typically shown a resistance to forgetting – perceptually salient utterance spoken by a female voice, independent of its semantic content, likely impairs remembering. Another finding suggests that females' superior capacity for processing the surface features of spoken utterances may predispose them to spontaneously employ effective strategies to retain content information despite distraction by perceptual features. This adds to the scant knowledge available on sex differences in not only directed forgetting, but also memory processing for vocal expressions, and underscores the importance of sex differences when processing spoken utterances in the directed-forgetting paradigm.

## Supporting Information

Appendix S1 **The list of voice samples used in the Experiment.**
(DOCX)
