# Supplementary material for: Her Voice Lingers on and Her Memory Is Strategic: Effects of Gender on Directed Forgetting
Source: PLoS One. 2013 May 15;8(5):e64030. doi: 10.1371/journal.pone.0064030 (PMC3655030; doi:10.1371/journal.pone.0064030)
Supplement: Appendix S1 — The list of voice samples used in the Experiment. (DOCX) [file pone.0064030.s001.docx]

**Appendix S1** The list of voice sample used in the Experiment.

| Stimuli | Voice Gender | Tone of Voice | Gender Accuracy | Tone Accuracy | Level of Angriness |
| --- | --- | --- | --- | --- | --- |
| Bucket | Female | Angry | 1.00 | 0.83 | 2.63 |
| Butter | Male | Neutral | 1.00 | 1.00 | 1.03 |
| Circle | Female | Angry | 1.00 | 0.90 | 2.77 |
| Detail | Female | Neutral | 0.97 | 0.97 | 1.07 |
| Effect | Male | Angry | 1.00 | 0.90 | 4.07 |
| Engine | Female | Neutral | 1.00 | 0.97 | 1.03 |
| Farmer | Male | Neutral | 1.00 | 1.00 | 1.13 |
| Fibre | Female | Angry | 0.90 | 0.90 | 3.03 |
| Finger | Female | Angry | 0.97 | 0.97 | 2.97 |
| Garlic | Male | Angry | 0.97 | 0.90 | 3.83 |
| Habit | Male | Neutral | 0.97 | 1.00 | 1.20 |
| Hammer | Female | Angry | 0.97 | 0.80 | 2.53 |
| Jacket | Male | Angry | 1.00 | 0.87 | 3.63 |
| Layer | Male | Neutral | 1.00 | 1.00 | 1.10 |
| Magnet | Female | Angry | 1.00 | 0.97 | 2.20 |
| Market | Female | Neutral | 1.00 | 0.97 | 1.00 |
| Number | Male | Neutral | 1.00 | 0.97 | 1.07 |
| Onion | Male | Angry | 1.00 | 0.93 | 4.13 |
| Pencil | Male | Neutral | 0.97 | 1.00 | 1.07 |
| Penny | Female | Angry | 0.90 | 0.83 | 2.90 |
| Plastic | Male | Angry | 1.00 | 0.97 | 3.87 |
| Printer | Female | Neutral | 1.00 | 0.97 | 1.03 |
| Rattle | Female | Neutral | 1.00 | 1.00 | 1.03 |
| Sailor | Female | Neutral | 1.00 | 1.00 | 1.00 |
| Seven | Male | Neutral | 1.00 | 0.97 | 1.07 |
| Signal | Male | Angry | 1.00 | 0.87 | 3.63 |
| Slipper | Male | Neutral | 0.97 | 1.00 | 1.03 |
| Tissue | Male | Angry | 1.00 | 0.87 | 3.80 |
| Vacuum | Female | Angry | 1.00 | 0.97 | 2.70 |
| Valley | Female | Neutral | 1.00 | 1.00 | 1.00 |
| Vision | Male | Angry | 1.00 | 0.90 | 3.70 |
| Window | Female | Neutral | 1.00 | 1.00 | 1.07 |

*Note.* Level of angriness was examined on a 5-point Likert scale anchored at 1=not at all and 5=very much.
